# Resource-Efficient Pet Dog Sound Events Classification Using LSTM-FCN Based on Time-Series Data

**DOI:** 10.3390/s18114019

**Published:** 2018-11-18

**Authors:** Yunbin Kim, Jaewon Sa, Yongwha Chung, Daihee Park, Sungju Lee

**Affiliations:** Department of Computer Convergence Software, Korea University, Sejong City 30019, Korea; kyb2629@korea.ac.kr (Y.K.); sjwon92@korea.ac.kr (J.S.); ychungy@korea.ac.kr (Y.C.); dhpark@korea.ac.kr (D.P.)

**Keywords:** pet dogs, separation anxiety, IoT sensor, sound events processing, resource efficiency, LSTM-FCN

## Abstract

The use of IoT (Internet of Things) technology for the management of pet dogs left alone at home is increasing. This includes tasks such as automatic feeding, operation of play equipment, and location detection. Classification of the vocalizations of pet dogs using information from a sound sensor is an important method to analyze the behavior or emotions of dogs that are left alone. These sounds should be acquired by attaching the IoT sound sensor to the dog, and then classifying the sound events (e.g., barking, growling, howling, and whining). However, sound sensors tend to transmit large amounts of data and consume considerable amounts of power, which presents issues in the case of resource-constrained IoT sensor devices. In this paper, we propose a way to classify pet dog sound events and improve resource efficiency without significant degradation of accuracy. To achieve this, we only acquire the intensity data of sounds by using a relatively resource-efficient noise sensor. This presents issues as well, since it is difficult to achieve sufficient classification accuracy using only intensity data due to the loss of information from the sound events. To address this problem and avoid significant degradation of classification accuracy, we apply long short-term memory-fully convolutional network (LSTM-FCN), which is a deep learning method, to analyze time-series data, and exploit bicubic interpolation. Based on experimental results, the proposed method based on noise sensors (i.e., Shapelet and LSTM-FCN for time-series) was found to improve energy efficiency by 10 times without significant degradation of accuracy compared to typical methods based on sound sensors (i.e., mel-frequency cepstrum coefficient (MFCC), spectrogram, and mel-spectrum for feature extraction, and support vector machine (SVM) and k-nearest neighbor (K-NN) for classification).

## 1. Introduction

Research on processing and analyzing big data in the IoT (Internet of Things) field has attracted considerable attention lately. In particular, parallel processing techniques, cloud computing technology, research for providing real-time services to users, and encryption have been actively investigated to find ways to more efficiently process large amounts of data [1,2,3,4,5]. Recently, an increase in the number of single-person households has led to studies on the behavior and control of companion animals, specifically the use of IoT sensor technology for the management of pet dogs. Research has been conducted on detecting dog behavior like sitting, walking, or running, to identify behavioral states [6] and actions like barking, growling, howling, or whining to identify emotional states [7]. For example, one study was conducted on pet dog health management that involved the detection of pet dog behaviors by means of acceleration sensors and heart rate sensors to identify food intake and/or bowel movements. Techniques have been developed for analyzing pet dog behavior to understand the emotional states, like depression or separation anxiety, of pet dogs who spend their time alone at home. With regard to understanding the emotions of pet dogs, sound events provide the most important information, which is why sound sensors are widely used [8]. In general, to classify the sound events, sound data are acquired by sound sensors, pre-processed in various ways to perform tasks like feature extraction, and then classified. Because data transfer and battery consumption are major issues for such sensors (sound and transmission sensors), which tend to have limitations on their capabilities, we need a way to classify pet dog sound events for resource-limited sensor devices. 

In this paper, we propose a way to efficiently classify pet dog sound events using intensity data and long short-term memory-fully convolutional networks (LSTM-FCN) based on time-series data. For this purpose, we acquire only intensity data by using a relatively resource-efficient noise sensor. In other words, the intensity data is acquired with an attachable noise sensor placed on a pet dog, and intensity sequences corresponding to barking, growling, howling, and whining are classified using a time-series data analysis method. It is difficult to attain sufficient classification accuracy using intensity data alone due to the loss of other information compared to sound data. To avoid significant degradation of classification accuracy, we apply LSTM-FCN, which is a method of deep learning analysis based on time-series data, and exploit the idea of bicubic interpolation. 

To verify the proposed method, actual pet dog sound events corresponding to barking, growling, howling, and whining were acquired from the internet, and the database was constructed using ground truth labels. Experimental results show that the proposed method based on noise sensor (i.e., Shapelet [9] and LSTM-FCN [10] for time-series) improves energy efficiency by 10 times without significant degradation of accuracy performance compared to typical methods based on sound sensor (i.e., MFCC [11], spectrogram [12], and mel-spectrum [13] for feature extraction, and SVM [14] and K-NN [15] for classification).

This paper is organized as follows. In Section 2, we summarize the time-series data classification problem and the LSTM-FCN approach that comprise the background of the proposed method. In Section 3, data acquisition, processing, and classification for intensity data obtained from attachable noise sensors are presented in detail. In Section 4, the experimental results are presented in terms of accuracy and energy efficiency. Finally, Section 5 discusses conclusions and future research. 

## 2. Background

### 2.1. Time-Series Classification

Time-series data can confirm trends in data between the past and the present, and time-series data is also sensitive to time-based information. Time-series data is largely found in domains that utilize real-time sensor data [16,17] such as traffic conditions [18,19], speech recognition [20,21], and weather information [22,23] using prediction and classification models [16,17,18,19,20,21,22,23]. In particular, a large amount of data flows from the sensor, and data warehouse technology [24] and techniques for analyzing this type of data are being developed. It is essential to convert the data into a meaningful form for accurate data analysis, which requires pre-processing the data before it can be used to develop a prediction or classification model. To improve classification accuracy, dimensional reduction [25,26,27] and data augmentation [28,29,30] have been studied. Garcke et al. [25] proposed a method to reduce the dimension of nonlinear time-series data extracted from wind turbines, setting the baseline so as to distinguish normal turbines from abnormal turbines, and monitoring the state of the wind turbines. In order to solve the multidimensional problem presented by time-series data acquired from a virtual sensor, dimension reduction was performed. In addition, Um et al. [28] proposed a method for applying convolutional neural networks (CNNs) to Parkinson’s disease data acquired from wearable sensors. To overcome the issue of using only a small amount of data, they improved classification accuracy by using various data augmentation methods like jittering, scaling, and rotation. In this way, dimension adjustment of the data is performed to compensate for missing data, to solve the “overfitting problem” in which accuracy is reduced due to excessive amounts of training data, and to address the “underfitting problem”.

### 2.2. LSTM-FCN

Time-series data is used in various fields to solve classification problems. Selecting a good classification model is important, as is acquiring high-quality data. Machine learning techniques such as hidden Markov models [31], dynamic time warping [32], and shapelets were developed to solve the time-series classification problem. LSTM-FCN is a recently developed method proposed by Karim et al. [10] to solve the time-series data classification problem. 

It consists of two blocks, a fully convolutional block and LSTM block, which receive the same time-series data. We use three convolutional layers composed of temporal convolutions to extract the characteristics of the input time-series data, and use batch normalization and the ReLU activation function to avoid vanishing gradients and exploding gradients during the learning process. Simultaneously, the LSTM block performs a dimensional shuffle on the received time-series data to convert it into a multivariate time-series with a single time step, which is processed by the LSTM layer. Finally, the multivariate time-series processed in each block is connected to a softmax classification layer, in which the time-series data can be classified.

In this paper, we acquired the intensity of sounds from the pet dogs using an attachable noise sensor. Each sound event was labeled as four sound event classes. In this case, since the sound event consisted of a sequence of varying dimensions, the dimension of the intensity data was transformed uniformly, and normalization and interpolation were performed to make the standard deviation of the values constant. To solve the problem, we apply LSTM-FCN to distinguish the time-series data after pre-processing each sound event.

## 3. Proposed Methods

Wearable devices for pet dogs require continuous data acquisition, since the behavior of the dog in the home is not limited to a certain period of time. In this paper, we propose a classification method for pet dog sound events using intensity data acquired by a relatively resource-efficient noise sensor (LM-393). The acquired data is time-series data in which the observation exhibits a pattern of temporal order. The data is labeled as the following four sound event classes: barking, growling, howling, and whining. After the acquired intensity data is transmitted over the wireless network via the IoT platform, classification is performed via pre-processing and feature extraction through the following four operations:

1. Labeling sound event as barking, growling, howling, or whining.
2. Applying normalization methods to obtain a constant data distribution.
3. Extending the dimension of learning data by interpolation.
4. Applying the LSTM-FCN model to classify the pet dog sound events.

**Figure 1:**
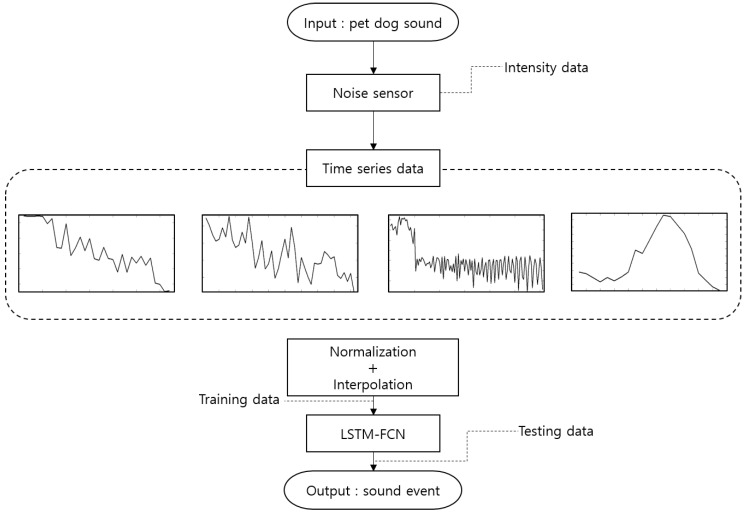
Overall structure of the proposed method.

Figure 1 shows the overall structure of the proposed method.

### 3.1. Pet Dog Sound Event Intensity Data Acquired by Noise Sensor

In this paper, intensity data corresponding to pet dog sound events are acquired using a noise sensor (LM-393) integrated with an Arduino sensor module. The noise sensor can amplify and control the sound generated by means of a variable resistor located on the upper portion of the sensor, if the sensitivity of the sound intensity is lower than desired. It senses sound based on this sensitivity and outputs it as voltage. The size of the sensor is 32 mm × 17 mm × 1 mm and the voltage is 3.3 V or 5 V. 

A wireless noise sensor is attached to the neck of the pet dog to obtain intensity data. When attaching such a noise sensor, the sensor and the dog’s neck strap must be finely adjusted to minimize noise caused by movement of the dog. The noise sensor outputs the intensity data over time at a rate of 138 data samples per second. The acquired intensity data is transmitted to the data storage device through Wi-Fi, after which each event is labeled as barking, growling, howling, or whining.

**Figure 2:**
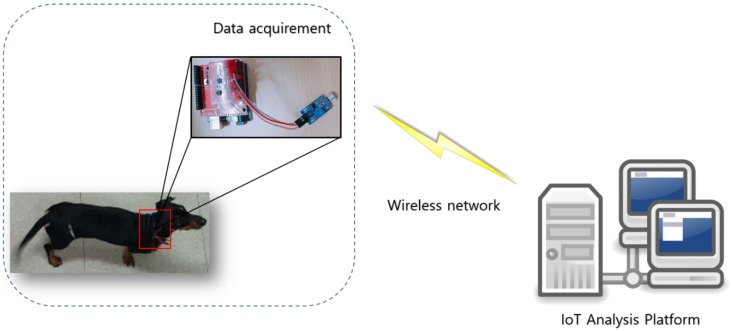
Intensity data acquisition using noise sensor. The noise sensors are used to collect intensity data and the collected intensity data is transmitted over the wireless network to the IoT analysis platform to process the data.

Figure 2 shows a noise sensor attached to the neck of a pet dog to acquire intensity data.

### 3.2. Analysis of Pet Dog Sound Intensity

**Figure 3:**
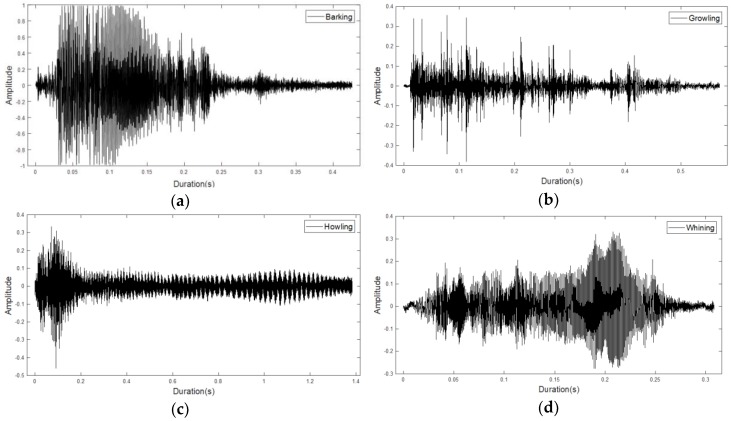
Waveforms for four pet dog sound events: (**a**) barking; (**b**) growling; (**c**) howling; (**d**) whining events.

Figure 3 shows plots of the sound data with the four sound features extracted from a sound sensor. When each sound event occurs (i.e., barking, growling, howling, or whining) the interval is set and extracted.

In the waveforms, we can see that the four classes have different characteristics. Figure 3a is the data corresponding to a general barking sound, and illustrates the frequency for approximately 0.4 s. Figure 3b is the “growling sound”, which exhibits a continuous signal. Figure 3c is characteristic of “howling sound”, which is a behavior by which pet dogs express loneliness. A strong waveform can be seen at the beginning of the sound, which decreases in the latter part. Figure 3d represents “whining sound”, a behavior that expresses fear and obedience, and is a representative example of howling to express separation anxiety. This exhibits a pattern similar to that of barking, but the amplitude is relatively low and the duration of the feature is short, with duration of approximately 0.1 s. Table 1 shows information on the sound data in Figure 3. Each field includes “CM” (CompressionMethod), which refers to the compression method used, “NC” (NumChannels), which is the number of audio channels encoded in the audio file, and “SR” (SampleRate), the sample rate of the audio data contained in the file. Additionally, the total number of samples “TS” (TotalSamples), the file playback time “Duration”, and the number of bits per sample “BPS” (BitsPerSample) encoded in the audio file are also included.

Note that the intensity data can be extracted from the sound data by treating the features as time-series data representing voltage information with the passage of time. At this time, the transmission option has 8 data bits, with the parity bit being set to “N”, and the stop bit being set to 1. Data is acquired at a rate of 138 samples per second. 

Although the LM-393 cannot provide exact sound intensity as well as sound data, the sound intensity level can be obtained by calculating the sound amplitude through “Peak to Peak” which means the minimum and maximum value among the changing voltages from the diaphragm. The diaphragm acquires electrical signal (analog voltage signal) with change of air pressure for sound in the audible frequency range. Finally, the continuous analog voltage signal is converted into digital data by using ADC (Analog to Digital Converter) with sampling, quantization, and encoding. Note that the ADC is built into the noise sensor, and the resolution is 10 bits (i.e., 2^10^ = 1024). Therefore, the noise sensor divides voltage signal from GND (Ground, 0 V) to VCC (Voltage of Common Collector, 5 V) by 10 bits resolution. The peak to peak represents the difference between these resolution ranges (0 to 1023), and then the calculated peak to peak data is converted to a value from 0 to 5 V (i.e., intensity level). In other words, with noise sensor, the intensity of the sound is measured by 1024 level (i.e., 1024 intensity level) with a value between 0 and 5 V [33].

**Figure 4:**
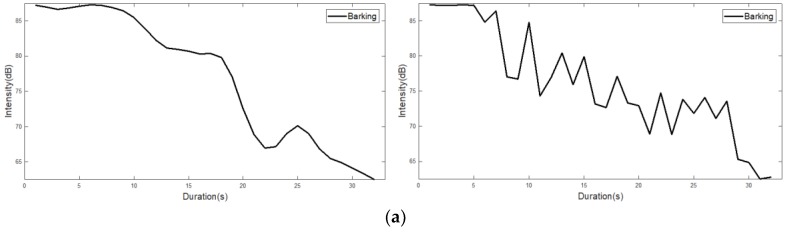

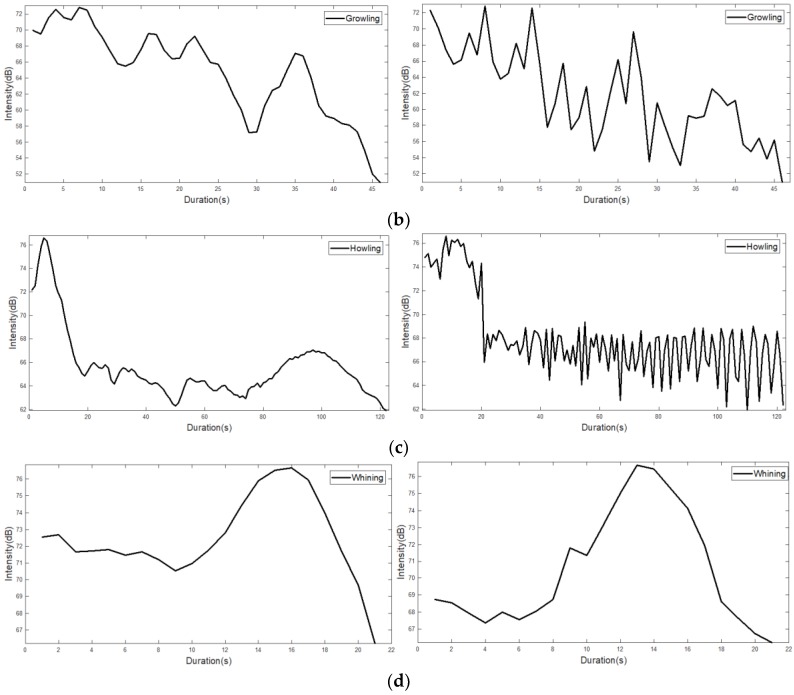
Intensity from the sound data and intensity level obtained from a noise sensor: (**left**) the intensity from the sound data; (**right**) the intensity level. (**a**) a barking event has a relatively short duration, and the value decreases rapidly after a certain period; (**b**) a growing event has a longer duration than the barking event, and also has a jagged characteristic; (**c**) a howling event shows the longest duration among the four sound events. It shows that the value of the early event is high and the value becomes low toward the rear part; (**d**) a whining event, such as barking, shows a short duration, and it also displays a jagged characteristic momentarily.

In Figure 4, left figures show the intensity from the sound data, and right figures shows the intensity level (i.e., noise intensity) obtained from a noise sensor. Although the noise sensor can measure the intensity level, it is difficult to obtain the accurate original intensity (i.e., sound intensity) as shown in Figure 4. To solve the problem, we exploited the idea of the interpolation technique without the difference of result in a significant change in the overall data. To compare sound intensity and noise intensity, we represent the intensity level as dB units, as shown in Figure 4. 

Figure 4 shows that the intensity level is shows a similar shape compared to the intensity. To find out the difference of intensity (i.e., sound sensor) and intensity level (i.e., noise sensor), we calculate the root mean square error (RMSE) with Equation (1), which is a generally used to measure the differences between values predicted by a model and the values observed. In Equation (1), $y_{i}$ and ${\hat{y}}_{i}$ are intensity and intensity level, respectively. Note that, the square root of the arithmetic average of the squared residuals of $y_{i}$ and ${\hat{y}}_{i}$ is statistically a standard deviation.

(1) $$\mathrm{RMSE}\;=\;\sqrt{\frac{1}{n}\overset{n}{\underset{i=1}{\sum }}{\left(y_{i}-{\hat{y}}_{i}\right)}^{2}}\;$$

Table 2 shows the results of RMSE between intensity and intensity level. With decreased RMSE, the similarity of sound intensity and noise intensity is increased. As shown in Table 2 with comparison of intensity (i.e., sound sensor) and intensity level (i.e., noise sensor), The RMES results of same sound events is relatively lower (i.e., barking-barking, growling-growling, howling-howling, and whining-whining are 4.61, 4.70, 3.54, and 3.13, respectively) than the different sound events (i.e., barking with growling, howling, and whining are 14.79, 8.63, 13.57, respectively). Therefore, the noise sensor can measure the intensity level, even if it is difficult to obtain the accurate original intensity.

The samples of intensity data acquired from the noise sensor have different lengths from the beginning to the end of the sound event. The length of the data can be used as a criterion in the feature extraction process, and if the data length is short, it may cause underfitting of the data. Table 3 shows the minimum, maximum, mean, and median lengths of the intensity data for each sound event.

The minimum length in Table 3 shows that both barking and whining sound events have a length of 5. The barking sound event has the lowest arithmetic mean at 19.24. The barking and whining sound events, which have relatively short lengths, experience considerable data loss relative to the original sound data. As described above, short data lengths present difficulties in extracting features to solve the classification problem.

Furthermore, since the sounds of pet dogs are different in size at the same sound event, the characteristics of size should be judged pointless. There is a problem that the range of the value is not constant because the intensity data acquired from the noise sensor outputs the value of the voltage by measuring the sound amplitude. This problem can lead to confusion by judging the magnitude of the value as minimum, maximum, mean, and median of intensity data. Table 4 shows the size comparison of the values of all the data sets acquired from the sensor.

To solve this problem, the ranges must be equal and the distributions must be similar. In this paper, we apply 0–1 normalization to achieve this. By using the maximum and minimum values of the voltage time-series data, the data can be transformed into a data set having an average distribution between 0 and 1. Equation (2) represents 0–1 normalization.

(2) $$\;Voltage=\;\frac{x_{voltage}-\min\left(x_{voltage}\right)}{\max\left(x_{voltage}\right)-\min\left(x_{voltage}\right)}\;$$

### 3.3. Bicubic Interpolation

In this paper, we apply anti-aliasing and interpolation to increase the data length without changing the features of the data. Interpolation is one of the image processing techniques used to acquire missing values among pixels when enlarging or reducing images. Especially, bicubic interpolation can be used in signal processing as well as image processing. It is performed by multiplying the values of the 16 adjacent vectors with weights based on their distance. This is advantageous in that interpolation can be performed naturally and accurately by obtaining the slope of the peripheral value and sampling the data. Bicubic interpolation is applied to the dataset obtained from the noise sensor to increase the amount of data by a factor of three. Equation (3) represents the process of bicubic interpolation:

(3) $$\;f\left(x,y\right)=\;\overset{3}{\underset{i=0}{\sum }}\overset{3}{\underset{j=0}{\sum }}a_{ij}x^{i}x^{i}.\;$$

**Figure 5:**
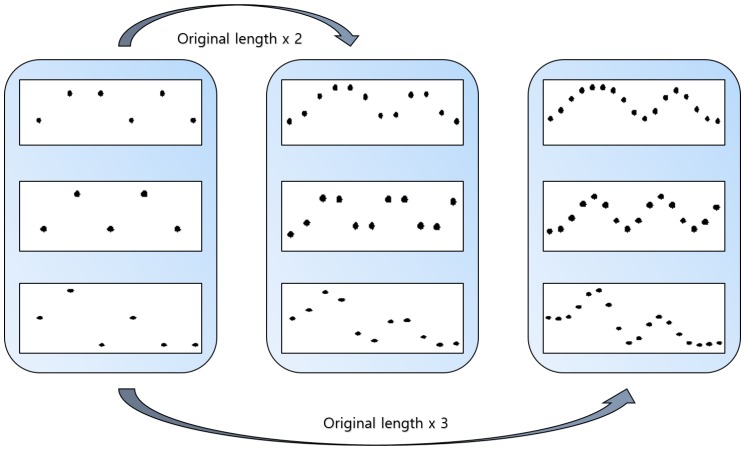
Sound events of increased length obtained via bicubic interpolation.

Figure 5 shows the change in the length of the time-series data after bicubic interpolation. It can be confirmed that the additional data produced by the bicubic interpolation demonstrates no meaningful change compared to the original data.

### 3.4. Classification of Pet Dog Sound Events Using LSTM-FCN

In this paper, we acquired intensity data for barking, growling, howling, and whining of pet dogs. The data were refined via bicubic interpolation, which is a traditional interpolation technique, and 0–1 normalization. We applied the LSTM-FCN method, which processes the input data through two networks, connects their results, and applies the softmax function. Seventy percent and 30% of the whole data were used in the learning process and evaluation process, respectively, of the LSTM-FCN. In other words, in 1200 intensity data samples, 840 comprised the training set, and the remaining 360 were used for the test set to verify the model. 

**Figure 6:**
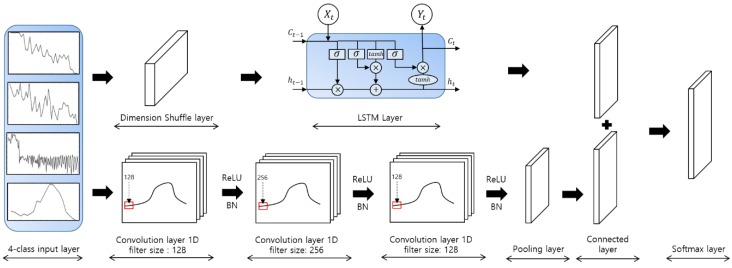
LSTM-FCN model for pet dog sound events classification.

Figure 6 shows the application of the LSTM-FCN model to the voltage time-series pet dog sound data. The filter sizes of the convolution layers were set to 128, 256, and 128, respectively, by default, and the ReLU activation function was used. The initial batch size was 128, the number of classes was 4, the maximum dimension was 647, and the number of epochs, which refers to the number of iterations required to learn all the data, was 2000.

The voltage time-series data represented as the four classes are converted from multivariate time-series data to single time step data by the dimension shuffle layer. The entire time-series data converted into a single time step are processed by the LSTM layer. Simultaneously, the same time-series data is shuffled through one-dimensional convolution layers with filter sizes of 128, 256, and 128 to perform fully convolutional network. This can be conducted in three steps, and the fully convolutional network of each step involves ReLU activation and batch normalization. By applying global average pooling, which outputs a feature map containing the reliability of the target class from the previous layer to the converted time-series data, the number of parameters of the network is reduced and the risk of overfitting is eliminated. The output values of the pooling layer and the LSTM layer are connected via the connected layer. Finally, the softmax is applied to allow for multiclass classification. At this time, the number of softmax layers is equal to the number of output layers.

Algorithm 1 shows the overall proposed method.
**Algorithm 1** Overall algorithm with the proposed method.Input: Intensity data obtained from pet dog sound event using noise sensor Output: Classification accuracy of pet dog sound event // *Load an intensity data* *Value* = Load (noise sensor) // *Normalization for uniform distribution* for (*I* = 0; *i* ≤ the number of columns in *Value*; *i*++)   for (*j* = 0; *j* ≤ the number of rows in *Value*; *i*++)     *Value_Normalize_*[*i,j*] = 0–1_Nomalization(*Value*[*i,j*]) // *Extending the dimension of learning data by interpolation* for (*I* = 0; *i* ≤ the number of columns in *Value*; *i*++)   for (*j* = 0; *j* ≤ the number of rows in *Value*; *i*++)     *Value_Intepolation_*[*i,j*] = BicubicInterpolation(*Value_Normailzation_*[*i,j*]) // *Classification of pet dog sound event using LSTM-FCN* for each *Value_Normailzation_*   Calculate accuracy of each pet dog sound event using *LSTM-FCN* Return;

## 4. Experimental Results

### 4.1. Experimental Environment

We conducted experiments using a noise sensor to classify the sound events of dogs using a single PC. The CPU of the utilized PC was an Intel Core i7-7700K (8 cores; Intel, Santa Clara, CA, USA), the GPU was an NVIDIA GeForce GTX 1080Ti 11 GB (3584 CUDA cores; NVIDIA, Santa Clara, CA, USA) and the RAM size was 32 GB. We also used TensorFlow 1.8 in Ubuntu 16.04.2 (Canonical Ltd., London, UK) to implement the LSTM-FCN technique and experimented with Keras, an open-source neural network library written in Python 3.6.5.

To acquire intensity data in a wireless environment, a noise sensor was connected to an Arduino Pro Mini board. The Arduino Pro Mini is the smallest available Arduino board and offers similar functionality as the ATmega328 series found in the usual Uno board. Furthermore, it is available as a 5 V/16 MHz model and 3.3 V/8 MHz model, which differ in their operating voltage and have input voltages of 5–9 V and 3.3–9 V, respectively. Since the proposed method involved attaching the sensor to the neck of the dog, a LM-393 noise sensor was used in combination with the Arduino Pro Mini 5 V/16 MHz. In addition, a Wi-Fi ESP8266 module was installed for wireless data transmission.

The intensity data acquired to classify the pet dog sound events were divided into four classes: barking, growling, howling, and whining. These were representative sounds produced by a pet dog in response to the stress of separation anxiety that can be felt by being isolated from the pet dog owner. Note that these sounds can also serve as an alert signal, or express fear in response to the presence of a stranger. Intensity data on the pet dog sounds were acquired via the attached noise sensor. The acquired intensity data were transmitted to the IoT analysis platform, which refined and processed the data.

The acquired data were classified according to four features, and each intensity data sample consisted of each sound event which was constituted as 300 datasets. For each feature, that is, the data generated from 300 sound events was labeled as one class, with a total of 1200 sound events. The pet dog sound events data sets are available in Supplementary Materials. Sampling of the acquired time-series data was performed at a rate of 138 samples per second, and thus we obtained a total of 88,617 samples.

Table 5 shows an example of intensity data for pet dog sound event obtained from noise sensor.

Table 6 shows the number of data for each class. Here, 5771 barking and 8390 whining events were acquired respectively due to their relatively short features. In addition, 17,877 growling and 56,579 howling events were obtained, respectively.

### 4.2. Comparison of Results Based on Sound and Intensity Data

In this paper, four sound events (i.e., barking, growling, howling, and whining) of pet dogs were acquired using a noise sensor. After that, 0–1 normalization was also applied to keep the distribution of data values constant. Then, we increased the lengths of the intensity data gradually with bicubic interpolation.

**Figure 7:**
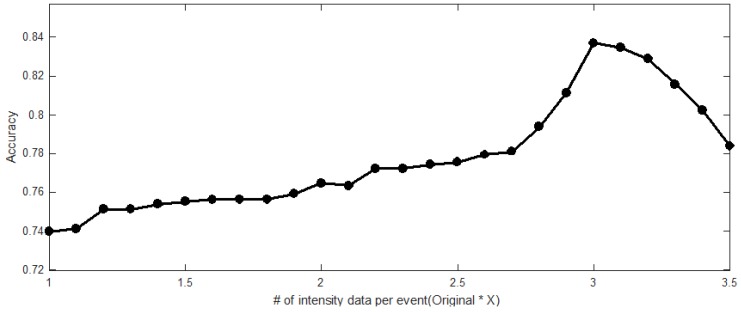
Each accuracy when increasing the data length through bicubic interpolation. The classification accuracy is improved if the length of the intensity data is increased compared to the original data. However, the classification accuracy is decreased if the increased length of the intensity data is exceeded three times compared to the length of the original data.

Figure 7 shows the visualization of each accuracy resulted in the LSTM-FCN model when the length of the datasets was increased through bicubic interpolation. The results show that the classification accuracy of the original data without bicubic interpolation is approximately 74%. When the length of the data was increased by a factor of three, we confirmed that the classification accuracy with bicubic interpolation was 84%. Note that the more length was increased than three times, the more classification accuracy was decreased.

In order to evaluate the proposed method, we conducted a comparative experiment on the sound data recorded by the sound sensor. The sound data recorded for the experiment were acquired irrespective of the type and size of the pet dogs. The sound data were obtained from uncompressed WAV (waveform audio file) format, which can convert analog sounds into digital without data loss. The sampling rate of the sound data was 44,100 Hz using a mono channel, and the data were not affected significantly by ambient noise. The 1200 acquired samples had the same duration.

To compared to typical approaches based on sound analysis, we used three feature extraction methods (i.e., MFCC [28], spectrogram [29], and mel-spectrum [30]) and two classification methods (i.e., SVM [31] and K-NN [32]). Note that, since the intensity data is time series data, we applied the Shapelets [33] and LSTM-FCN [27] without the feature extraction methods. Note that, to extract the features, each the pet dogs sound corresponding to the interval of 1 to 3 s was separated manually.

Table 7 compares each accuracy of using different features (MFCC, Spectrogram, and mel-spectrum) and classification methods (SVM, K-NN, Shapelet, LSTM-FCN, and LSTM-FCN with bicubic). To validate the proposed method, a comparative experiment was performed using the time-series methods (i.e., Shapelet, LSTM-FCN, and LSTM-FCN with bicubic) on the intensity data and the typical method (i.e., SVM and K-NN). The accuracies of the typical classification methods were approximately 78% to 86%, versus approximately 74% for the LSTM-FCN and 84% for the LSTM-FCN with bicubic interpolation. These results confirm that the proposed method is suitable for classifying the sound events of a pet dog. Although, the proposed method achieves relatively low accuracy compared to the typical methods, it manages to attain a high gain in energy efficiency, including data size and power consumption without degradation of significant accuracy.

Table 8 lists the data size and power consumption of the sound sensor, noise sensor, and Wi-Fi sensor used in the experiment. The power of the noise sensor represents the sum of the power of the Arduino Pro Mini and the LM-393 sensor.

With regard to the average data size, the sound data sensor performs relatively poorly due to the nature of the WAV format, which uses no compression. Therefore, the intensity data achieves a value approximately 73.8 times better than that of the sound data in this regard. In addition, the supply voltage of the sound sensor and the noise sensor used in the experiment is 5 V, which means that the same voltage value is applied to both. The difference in current can be attributed to differences in overall resource efficiency.

In addition, the proposed method utilizes a system whose efficiency is sensitive to the battery usage time. The efficiency, with respect to battery usage time, is calculated based on the capacity of a Li-ion battery installed in a typical wearable device such as a smart watch. To date, no smart watch has been released that exceeds 400 mAh. This is one of the disadvantages of wearable devices that result from miniaturization. For example, when the battery capacity was 400 mAh, and the voltage was 5 V, the total amount of electrical energy was 7200 J. Since the sensing data has to be transmitted to the IoT platform, the transmission energy consumption should be also considered. To calculate the transmission energy consumption, we used 802.11b, which was supported by Wi-Fi sensor. The 802.11b standard technology has a theoretical maximum transmission rate of 11 Mbps, and supports a transmission speed of about 6 to 7 Mbps in the implementation of CSMA/CA technology. Therefore, we used network conditions with 300 KB/s to 1200 KB/s as shown in Table 7. To calculate the total energy consumption, we considered both the sensing and transmission energy consumption. Note that, the transmission energy consumption depends on the network conditions. Since the sound sensing data required lager transmission data size than noise sensing data, the transmission energy consumption was also more required.

The sensing energy consumption of sound and noise was 0.9 J and 0.1 J for one second with various network conditions, respectively, and the transmission energies of 0.111 to 0.028 J and 0.002 to 0.001 J were obtained. Finally, by calculating the battery usage time (i.e., battery capacity was 400 mA), we found out that the sound sensor can be used for about 1.9 h, and the noise sensor can be used for 19.6 h. Therefore, we confirmed that the proposed method (i.e., with noise sensor) can improve the energy efficiency about 10 times than the typical method (i.e., with sound sensor). Table 9 shows that sensing, transmission, total energy consumption for one second, and battery usage time with various network conditions (i.e., 300, 600, 900, and 1200 KB/s).

## 5. Conclusions

The classification of pet dog sound events using data from a sound sensor is important for analyzing the behavior or emotions of pet dogs that are left alone. In this paper, we proposed a way to classify pet dog sound events (barking, growling, howling, and whining) to improve resource efficiency without significant degradation of accuracy. We acquired intensity data from pet dog sound events using a relatively resource-efficient noise sensor instead of a sound sensor. Generally, it is difficult to achieve sufficient classification accuracy using the intensity of sound, due to the loss of information in the sound data. To avoid significant degradation of classification accuracy, we applied LSTM-FCN, and exploited bicubic interpolation. Based on the experimental results, which found the typical methods to be 78% to 86% accurate and the proposed method to be 84% accurate, we can confirm that the proposed method based on noise sensor based on noise sensor (i.e., Shapelet and LSTM-FCN for time-series) improved energy efficiency by 10 times without significant degradation of accuracy compared to typical methods based on sound sensor (i.e., MFCC, Spectrogram, and mel-spectrum for feature extraction, and SVM and K-NN for classification).

## Figures and Tables

**Table 1:** Information on pet dog sound data file format.

Pet Dog Sound Events	Field Name					
	CM	NC	SR	TS	Duration	BPS
Barking	Uncompressed	1	22,050	5327	0.24	16
Growling	Uncompressed	1	22,050	11,461	0.51	16
Howling	Uncompressed	1	22,050	32,628	1.47	16
Whining	Uncompressed	1	22,050	6311	0.28	16

**Table 2:** The results of RMSE between intensity and intensity level.

Pet dog Sound Events		Noise Sensor (Intensity Level)			
		Barking	Growling	Howling	Whining
Sound sensor (Intensity)	Barking	4.61	14.79	8.63	13.57
	Growling	10.41	4.70	8.14	8.34
	Howling	8.89	8.89	3.54	7.93
	Whining	9.57	8.38	7.81	3.13

**Table 3:** The minimum, maximum, mean, and median lengths of the intensity data for each sound events.

Pet dog Sound Events	Field Name			
	Minimum Length	Maximum Length	Mean Length	Median Length
Barking	5	47	19.24	19
Growling	16	405	59.59	56
Howling	51	646	188.60	161
Whining	5	198	27.97	19

**Table 4:** Differences of voltage value according to each sound event of data extracted from sensor.

Pet Dog Sound Events	Field Name			
	Minimum Voltage	Maximum Voltage	Mean Voltage	Median Voltage
Barking	0.98	25.39	15.70	17.58
Growling	0.98	8.79	6.29	5.86
Howling	0.98	11.72	7.68	7.81
Whining	0.98	13.67	8.32	7.81

**Table 5:** An example of intensity data for pet dog sound event obtained from noise sensor.

Pet Dog Sound Events	1/138 Sec	Intensity Level															
Barking	1–16	4.82	4.81	4.79	4.81	4.81	4.34	4.66	4.46	4.47	4.19	3.48	2.84	2.23	1.68	1.59	2.38
	17–32	2.84	3.62	4.34	3.92	2.97	2.31	2.28	2.54	2.82	3.09	3.38	3.50	3.26	2.85	2.63	2.84
	33–48	2.23	1.68	1.59	2.38	3.62	4.34	3.92	2.97	2.31	2.28	2.54	2.82	3.09	3.38	3.50	3.26
	49–64	2.85	2.63	2.84	3.23	3.4	3.07	2.52	1.94	1.91	2.16	2.22	2.55	2.85	3.00	3.11	3.33
Growling	1–16	4.45	4.28	3.83	2.99	2.49	2.63	3.11	3.54	3.80	4.01	4.04	3.73	3.26	2.93	2.94	3.11
	17–32	3.17	2.96	2.64	2.37	2.14	1.96	2.03	2.58	3.37	3.91	3.85	3.52	3.39	3.74	4.28	4.55
	33–48	4.29	3.76	3.22	2.66	2.09	1.81	2.01	2.49	2.94	3.25	3.53	3.66	3.48	3.15	3.06	3.49
	49–64	4.15	4.50	4.25	3.70	3.14	2.57	1.99	1.65	1.68	1.94	2.25	2.63	3.05	3.19	2.77	2.03
Howling	1–16	3.61	4.08	4.28	4.15	3.95	3.52	2.64	2.18	1.95	1.88	2.01	2.47	3.13	3.58	3.56	3.32
	17–32	3.19	3.31	3.54	3.75	3.88	3.99	4.03	3.92	3.75	3.65	3.77	2.55	2.15	2.62	2.57	2.08
	33–48	2.48	3.30	3.89	3.97	3.81	3.06	2.48	2.20	2.54	3.18	3.54	3.27	2.73	2.37	2.38	2.57
	49–64	2.80	3.11	3.45	3.50	2.93	2.08	1.55	1.71	2.19	2.50	2.35	2.03	1.82	1.85	2.00	2.11
Whining	1–16	0.01	0.33	0.76	1.05	1.04	0.89	0.71	0.48	0.19	0.76	1.41	2.20	2.62	2.27	1.56	1.11
	17–32	1.69	1.98	2.16	2.96	3.16	3.31	3.41	3.46	3.47	3.35	3.02	2.57	2.20	2.01	1.85	1.65
	33–48	1.22	0.70	0.31	0.10	0.01	0.15	0.39	0.52	0.40	0.18	2.20	2.54	3.18	3.54	2.73	2.37
	49–64	2.08	1.94	1.81	1.63	1.44	1.38	1.57	1.90	2.09	1.97	1.72	1.58	1.94	2.21	1.95	1.68

**Table 6:** Number of data of four pet dog sound events.

Pet Dog Sound Events	# of Events	# of Intensity Data per Event
Barking	300	5771
Growling	300	17,877
Howling	300	56,579
Whining	300	8390
Total Number of Data	1200	88,617

**Table 7:** Classification accuracy applied to each model.

Type of Sensor	Sound Sensor (Typical)						Noise Sensor (Proposed)		
Type of Data	Sound data						Intensity Data		
Feature Extraction Method	MFCC		Spectrogram		Mel-Spectrum		None		
**Classification method**	SVM	K-NN	SVM	K-NN	SVM	K-NN	Shapelet	LSTM-FCN	Bicubic + LSTM-FCN
**Accuracy**	0.8545	0.7944	0.8633	0.7834	0.8432	0.7855	0.6788	0.7396	0.8368

**Table 8:** Comparison of data size and performance between sound sensor and noise sensor, and Wi-Fi sensor.

Type of Device	Average of Data Size (KB)	Current (mA)	Voltage (V)	Energy (J)
Sound sensor (MQ-U300)	66.4	180	5.0	0.9
Noise sensor (LM-393)	0.9	20	5.0	0.1
Wi-Fi (ESP8266)	—	170	3.3	0.5

**Table 9:** Comparison of energy consumption and battery usage with various network conditions.

		Transmission Speed (KB/s)			
		300	600	900	1200
Sensing Energy (J)	Sound	0.9			
	Noise	0.1			
Transmission Energy (J)	Sound	0.111	0.056	0.037	0.028
	Noise	0.002	0.001	0.001	0.001
Total Energy (J)	Sound	1.011	0.956	0.937	0.928
	Noise	0.102	0.101	0.101	0.101
Battery usage time (h)	Sound	1.9	2.0	2.1	2.2
	Noise	19.6	19.8	19.8	19.8

## Supplementary Materials

The pet dog sound events data sets are available online at https://github.com/kyb2629/pdse.
